# Expanding the Scope of Aluminum Chemistry with Noninnocent Ligands

**DOI:** 10.1021/acs.accounts.3c00714

**Published:** 2024-04-06

**Authors:** Leo W. T. Parsons, Louise A. Berben

**Affiliations:** Department of Chemistry, University of California, Davis, California 95616, United States

## Abstract

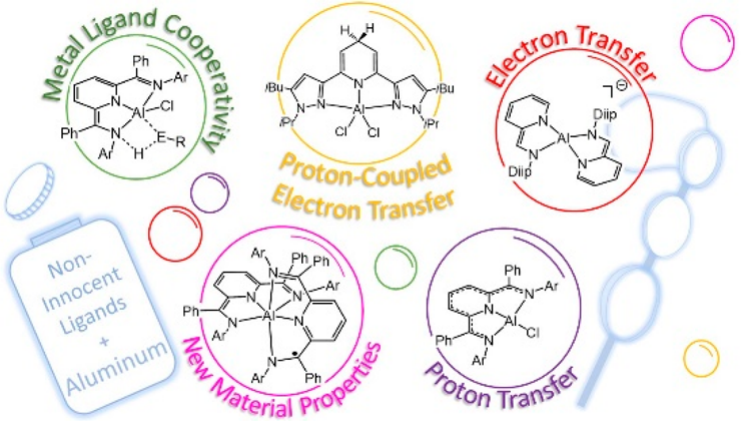

Aluminum is the most abundant metal in the earth’s crust at 8%, and it is also widely available domestically in many countries worldwide, which ensures a stable supply chain. To further the applications of aluminum (Al), such as in catalysis and electronic and energy storage materials, there has been significant interest in the synthesis and characterization of new Al coordination compounds that can support electron transfer (ET) and proton transfer (PT) chemistry. This has been achieved using redox and chemically noninnocent ligands (NILs) combined with the highly stable M(III) oxidation state of Al and in some cases the heavier group 13 ions, Ga and In.

When ligands participate in redox chemistry or facilitate the breaking or making of new bonds, they are often termed redox or chemically noninnocent, respectively. Al(III) in particular supports rich ligand-based redox chemistry because it is so redox inert and will support the ligand across many charge and protonation states without entering into the reaction chemistry. To a lesser extent, we have reported on the heavier group 13 elements Ga and In, and this chemistry will also be included in this Account, where available.

This Account is arranged into two technical sections, which are (1) Structures of Al–NIL complexes and (2) Reactivity of Al–NIL complexes. Highlights of the research work include reversible redox chemistry that has been enabled by ligand design to shut down radical coupling pathways and to prevent loss of H_2_ from unsaturated ligand sites. These reversible redox properties have in turn enabled the characterization of Class III electron delocalization through Al when two NIL are bound to the Al(III) in different charge states. Characterization of the metalloaromatic character of square planar Al and Ga complexes has been achieved, and characterization of the delocalized electronic structures has provided a model within which to understand and predict the ET and PT chemistry of the NIL group 13 compounds. The capacity of Al–NIL complexes to perform ET and PT has been employed in reactions that use ET or PT reactivity only or in reactions where coupled ET/PT affords hydride transfer chemistry. As an example, ligand-based PT reactions initiate metal–ligand cooperative bond activation pathways for catalysis: this includes acceptorless dehydrogenation of formic acid and anilines and transfer hydrogenation chemistry. In a complementary approach, ligand based ET/PT chemistry has been used in the study of dihydropyridinate (DHP^–^) chemistry where it was shown that N-coordination of group 13 ions lowers kinetic barriers to DHP^–^ formation. Taken together, the discussion presented herein illustrates that the NIL chemistry of Al(III), and also of Ga(III) and In(III) holds promise for further developments in catalysis and energy storage.

## Key References

- ArnoldA.; SherbowT. J.; SaylerR. I.; BrittR. D.; ThompsonE. J.; MunozT. M.; FettingerJ. C.; BerbenL. A.Organic electron delocalization modulated by ligand charge states in [L_2_M]^*n*−^ complexes of group 13 ions. J. Am. Chem. Soc.2019, 141, 15792–1580331510741 10.1021/jacs.9b05602.^1^*Our initial report of octahedral I_2_P complexes of Al(III), Ga(III), and In(III), where two tridentate ligands bind the group 13 ion: mixed-valent, diradical, and other electronic properties were characterized.*
- BassT. M.; CarrC. R.; SherbowT. J.; FettingerJ. C.; BerbenL. A.Synthesis of Square Planar Gallium Complexes and a Proton NMR Correlation Probing Metalloaromaticity. Inorg. Chem.2020, 59, 13517–1352332883068 10.1021/acs.inorgchem.0c01908.^2^*An experimental method to probe the involvement of group 13 3+ ions in the overall aromaticity of the molecule is described along with the first reported square-planar Ga complexes.*
- CarrC. R.; VestoJ. I.; XingX.; FettingerJ. C.; BerbenL. A.Aluminum–ligand cooperative O–H Bond Activation Initiates Catalytic Transfer Hydrogenation. ChemCatChem.2022, 14, e202101869.^3^*Al–ligand cooperative bond activation initiates transfer hydrogenation of benzophenone to diphenylmethane with isopropanol H_2_ donor.*

## 1 Introduction

Recent decades have seen extensive research and development in main-group reaction chemistry, where accessible oxidation states and bonding modes have been greatly expanded as a result of efforts in synthetic chemistry.^4,5^ The element aluminum, Al, has many features that suggest it would be appealing for large-scale processes if its chemistry were developed in a way that enabled those applications. For example, Al has a very high natural abundance; with the earth’s crust comprising 8% Al, it is the third most abundant element.^6^ It is domestically available in many countries and provides security in supply chains. Also, Al is relatively inexpensive with a current price of just $2,213/ton.^7^ The purification and extraction of Al are notoriously high-energy processes driven electrochemically, but recent innovations have both lowered the energy inputs through advances in process engineering and reduced their costs with renewably derived electricity.

This Account focuses on the chemistry of noninnocent ligand (NIL) Al complexes, particularly how electronic structures and reaction pathways can be expanded using NILs. To a lesser extent, this Account will also discuss the NIL chemistry of gallium, Ga, and indium, In, where the comparison is useful. Both the lightest and the heaviest of the group 13 elements, boron, B, and thallium, Tl, respectively, have not been characterized as M(III) ions with NILs, to the best of our knowledge. We begin with a brief overview of the known NIL chemistry of Al. Sections 1.1 and 1.2 include strategies for the syntheses of iminepyridine–Al compounds with reversible electron transfer (ET) and proton transfer (PT) chemistry. Section 1.3 summarizes and explains some of the electronic properties of the iminepyridine–Al complexes that can be accessed. The reaction chemistry of NIL–Al complexes can be categorized into three main types, as outlined in Section 2: ET, PT, and proton-coupled ET (PCET) reactions. The subsections discuss each of these reaction types. Section 2.1 describes ligand ET chemistry; section 2.2 describes Al–ligand cooperative bond activation for catalysis, which is enabled by ligand PT chemistry; and section 2.3 focuses on ligand-based PT coupled with ET chemistry, which is also organohydride chemistry. The reader is also referred to several excellent reviews on the coordination chemistry of Al, which have focused on the synthesis of coordination compounds,^8−11^ Al-mediated catalysis,^12−17^ and metal–ligand cooperative bond activation.^18,19^ This Account contains very little overlap with those prior reports.

**Scheme 1:**
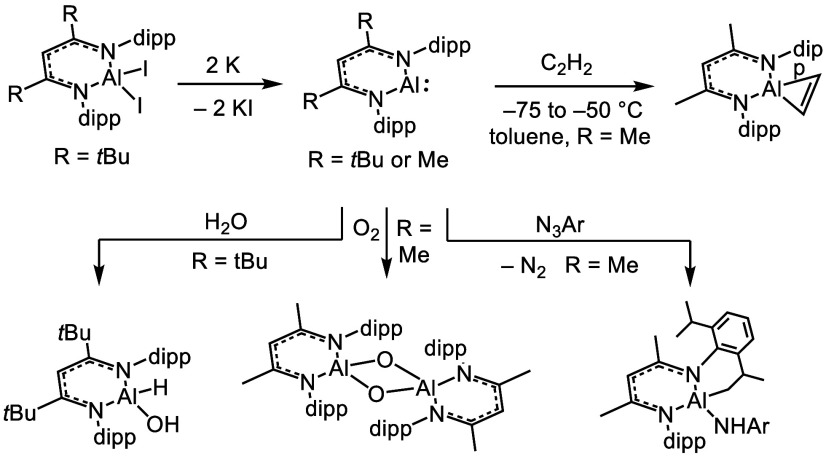
Reaction Chemistry Based on the Al(I/III) Couple, Which Includes Oxidative Addition Chemistry dipp = 2,6-diisopropylphenyl (2,6-*i*Pr_2_Ph).

ET and PT elementary reaction steps are common to many chemical transformations, but they are difficult to accomplish with many of the main-group elements, including Al. Redox cycling through Al(I) and Al(III) requires significant energy input and accesses highly unstable and air-sensitive compounds of Al(I). Despite these difficulties, several elegant reports of redox chemistry mediated by the Al(I/III) couple have been reported (Scheme 1).^20,21^ In these examples, a β-diketiminate ligand supports both the Al(I) and Al(III) redox states without participating directly in the redox chemistry via ligand-based ET.

### 1.1 Overview of NILs That Have Been Reported with Al

**Scheme 2:**
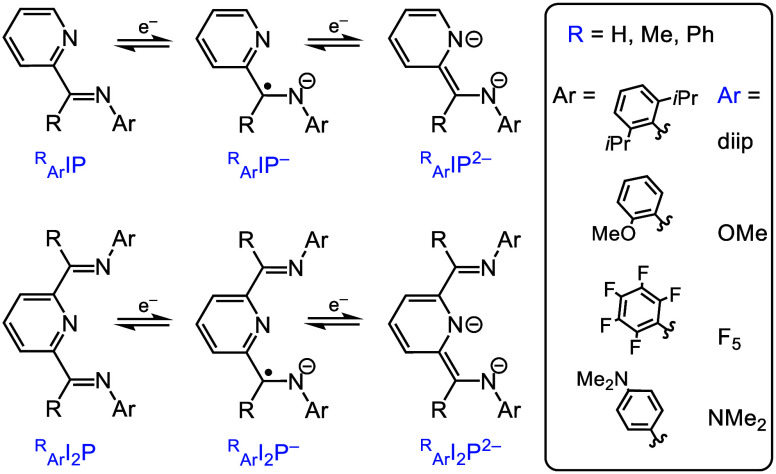
NILs That Have Been Studied with Al(III) Include Iminepyridine (IP) and Diiminepyridine (I_2_P), Substituted by R and Ar groups, Which Are Denoted in Blue Ligands are shown here with the commonly observed charge states that are accessible with group 13 ions, Al(III) and Ga(III).

**Scheme 3:**
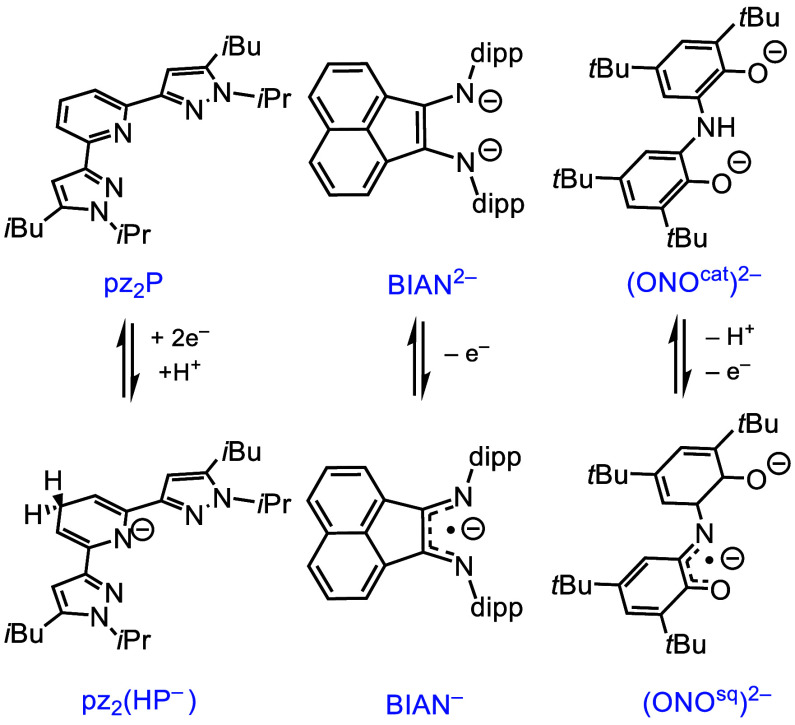
NILs That Have Been Studied on Al(III) Include Dipyrazoylylpyridines (pz_2_P), Acenaphthene-1,2-diimines (BIAN), and Bis(3,5-di-*tert*-butyl-2-phenol)amine (ONO) cat and sq superscripts are catecholate and semiquinonate forms of the ONO ligand. Charge states shown are common when coordinated to group 13 ions, Al(III) and Ga(III).

The chemistry of NILs is an area of research that continues to develop, and it expands the chemistry of metal centers across the periodic table, by making more of their electronic states accessible.^22−26^ Some examples of NILs that have been characterized with Al(III) are highlighted in this section as an introduction to the remainder of the Account. Iminepyridine (abbreviated as IP, Scheme 2) was initially reported in complexes with Mg, and diiminepyridines (abbreviated as I_2_P, Scheme 2) were reported in metal–ligand complexes with first-row transition elements by Wieghardt and co-workers.^27^ Main-group I_2_P complexes first appeared with Ga, in work by Richeson and co-workers.^28^ Work by Chirik in this area has developed the reaction chemistry of transition-element I_2_P complexes, which includes the 2 + 2 cycloadditions of dienes and olefin catalysis, among many others.^29,30^ Other NILs that have been studied with Al(III) include dipyrazolylpyridines (generally abbreviated here as pz_2_P), acenaphthene-1,2-diimines (BIAN), and bis(3,5-di-*tert*-butyl-2-phenol)amine (ONO) (Scheme 3); the chemistry of these NILs will be mentioned in the appropriate sections of this Account.

### 1.2 Synthetic Strategies for Iminepyridine–Al compounds with Reversible ET and PT Chemistry

**Scheme 4:**
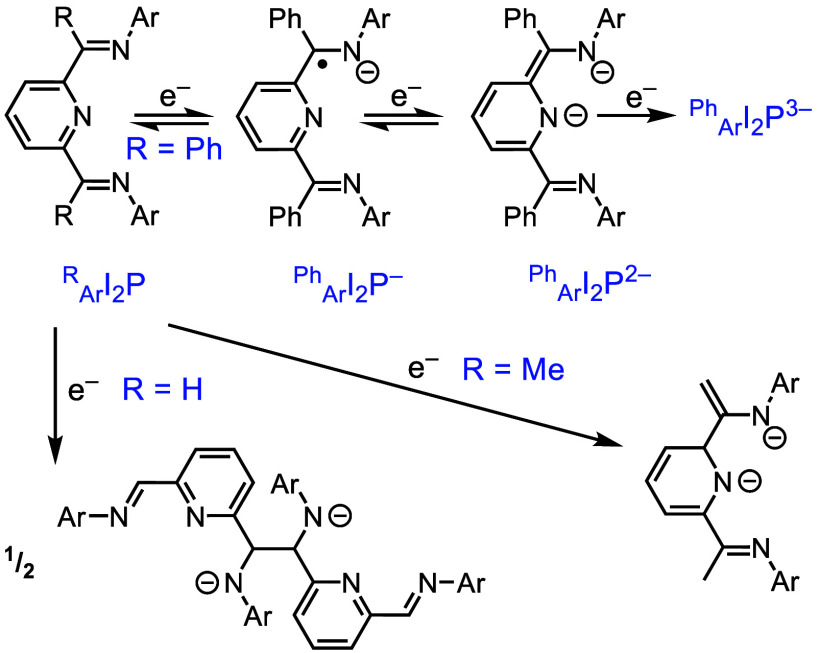
Commonly Observed Reactivity of Substituted _Ar_^R^I_2_P Ligands upon One-Electron Reduction

Although not specifically mentioned above, the foregoing examples of I_2_P-supported transition-element chemistry employ primarily I_2_P ligands that are Me substituted at the imine C atom.^23,22,27,29,30^ This is denoted in the ligand abbreviation as a preceding superscript when needed (Scheme 2). Substituents on the imine N atom can be denoted as a preceding subscript. For example, the most commonly used I_2_P ligand has Me on the imine C atoms and dipp (defined in Scheme 1 and 2) on the imine N atoms: _dipp_^Me^I_2_P. The analogous ligand with only H on the imine C, _dipp_^H^I_2_P, is rare in the literature, likely because of the potential for radical C–C coupling (Scheme 4). When coordinated to a metal ion (transition metal or group 13), ^Me^I_2_P ligands commonly lose H_2_ in a chemically irreversible process, and the resulting ^CH_2_^I_2_P ligand form can support extensive metal-based chemistry, as described in section 1.1.

When discussing the chemistry of _Ar_^Ph^I_2_P in this Account, we often leave off the superscript of the understood phenyl group (Scheme 4). To simplify notation, similarly, the Ar group on the imine N is commonly dipp and is also left off. Exceptions are when variation of the Ar group is a key aspect of the work, and subscripts are then included. Conveniently, all the I_2_P derivatives can be easily modified at the imine N. Ar groups are incorporated via a condensation reaction with aryl amines as the final step in I_2_P synthesis. Larger Ar groups, such as dipp in _dipp_I_2_P, support the formation of 1:1 ligand–Al complexes. Smaller substituents at the 2 and 6 positions of the aryl ring (e.g., 2-MeOPh) support the formation of 2:1 ligand–Al complexes, which are coordinatively saturated and six-coordinate at the Al center.

**Scheme 5:**
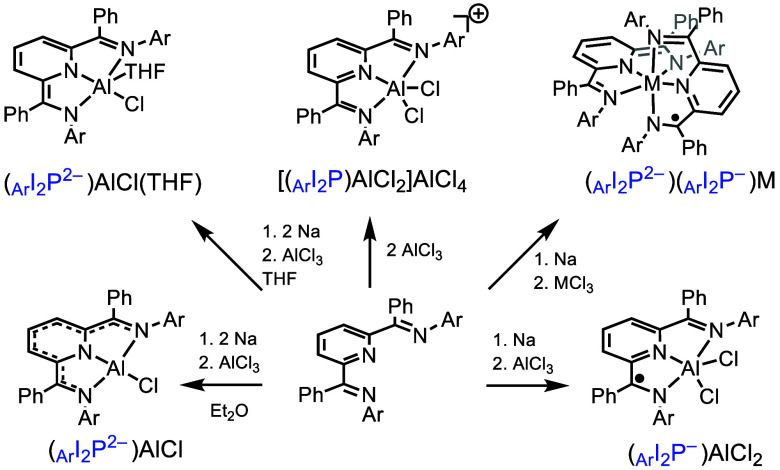
Synthetic Roadmap to _Ar_I_2_P–Al Complexes Reported by Our Lab with Ligands in Various Charge States, M = Al, Ga, or In For singly ligated Al complexes, Ar = dipp; for doubly ligated octahedral complexes, Ar = o-PhOMe, PhF_5_, or p-PhNMe_2_, as in Scheme 2.

Neutral ligand complexes are commonly prepared by the addition of two equivalents of a group 13 metal chloride, MCl_3_, to one equivalent of a neutral ligand, L, to produce a salt of the form (LMCl_2_)(MCl_4_)^2,31^ (Scheme 5). Reduced ligand complexes can then sometimes be prepared by reduction of the (LMCl_2_)(MCl_4_) salt with chemical reductants.^2,32^ As examples, _dipp_I_2_P complexes of Al can be synthesized by addition of two equivalents of AlCl_3_ to one equivalent of _dipp_I_2_P, which affords [(_dipp_I_2_P)AlCl_2_](AlCl_4_). In a second common approach, reduced ligand complexes are prepared by reduction of the ligand with an alkali-metal reductant. For example, Na can be used to afford Na^+^L^–^ or (Na^+^)_2_L^2–^, which can then undergo salt metathesis with MCl_3_ to afford NaCl and (L^*n–*^)MCl_(3–*n*)_.^33^ In some instances, a one-pot method involving a mixture of L, MCl_3_, and a reductant is more effective for the formation of (L^*n–*^)MCl_(3–*n*)_, as in the syntheses of complexes with the fluorinated _PhF_5__I_2_P ligands (introduced in Scheme 2).^34^ A third common synthetic approach is where the starting ligand is already isolated in a reduced and protonated state: in that case, deprotonation by a base such as NaH or KH affords the alkali-metal ligand salt, which will react with MCl_3_. This deprotonation approach has been employed in the synthesis of bis(enol)amine,^35^ and in the synthesis of Al complexes with an iminocatecholate pincer ligand,^36^ as examples. The three approaches described in this section have been employed extensively across many examples. However, there are some examples in which radical ligand intermediates generated by the reduction reactions are unstable toward dimerization,^37,38^ or decomposition. Ligand design can play an important role in the successful metalation of NILs, such as the inclusion of steric bulk to physically block radical reactions^39^ or the addition of stabilizing organic functional groups like phenyl in place of an H atom. Once obtained, NIL complexes can sometimes be oxidized or reduced to obtain another charge state in a series of compounds.

Solvent choice plays a role in the outcomes of the reactions described above. More polar solvents, such as tetrahydrofuran (THF) or dimethoxyethane (DME), promote faster ligand reduction than diethyl ether (Et_2_O) or alkane or aromatic solvents. Coordinating solvents, such as THF, may also coordinate to the metal. An example here is the formation of five-coordinate (_dipp_I_2_P^2–^)AlCl(THF) and four-coordinate (_dipp_I_2_P^2–^)AlCl; they are obtained from the same reaction performed in THF or Et_2_O, respectively.^35,40^ When the group 13 ion is coordinatively saturated, as in octahedral complexes with tridentate ligands, then some kinetic stability toward strongly donating solvents, such MeCN and H_2_O, has been observed, but these are rare examples.^1,34^

### 1.3 Electronic Properties of NIL–Al Complexes

While the unique reactivity afforded by NILs bound to Al(III) [or Ga(III)] is the main subject of this Account, this reactivity can sometimes be rationalized or predicted if an appreciation of the electronic structures of the compounds is presented first. The metal–ligand bonding has covalent character between the metal valence p-orbitals and the ligand-based p-orbitals that make up the π-bonding in the ligands. Those interactions impart properties, such as metalloaromaticity, exchange coupling pathways, and Class III delocalization, and they enable tuning of the Lewis acidity of the Al(III) or Ga(III) ion by modification of the I_2_P ligand’s electronic structure.

**Chart 1:**
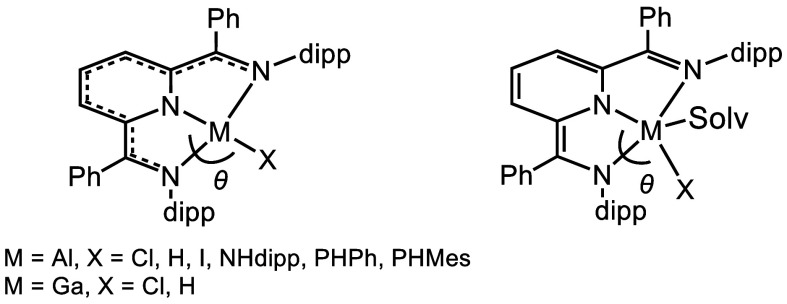
Four-Coordinate Compounds (left) and the Five-Coordinate Solvated Analog (right)a

We first consider the geometric and electronic structures of the square planar (SP) Al and Ga complexes (I_2_P^2–^)AlCl, (I_2_P^2–^)AlH, (I_2_P^2–^)AlI, (I_2_P^2–^)GaCl, and (I_2_P^2–^)GaH (Chart 1).^2^ The other four-coordinate molecules, (I_2_P^2–^)Al(NHdipp),^41^ (I_2_P^2–^)Al(PHPh), and (I_2_P^2–^)Al(PHMes) (Chart 1)^42^ are distorted from SP. NHdipp, PHPh, and PHMes are 2,6-diisopropylphenylamide, phenylphosphide, and mesitylphosphide, respectively. The NIR absorption spectra for all of these compounds display broad and intense electronic transitions around 1000 nm, which have been assigned as ligand–metal charge transfer (LMCT) bands because the reduced ligands donate electron density into the metal-based p-orbitals. Observations from single-crystal X-ray diffraction studies suggest that the coordination of the monodentate ligands to the metal center influence the electronic structures of the I_2_P ligand. Four-coordinate metal centers have a delocalized I_2_P electronic structure, and five-coordinate metal centers show alternating single- and double-bond character (Chart 1). Another interesting feature of all the four-coordinate group 13 structures is the N_py_–M–X angle (where N_py_ is the pyridyl N and X is a monodentate ligand), which deviates further from θ = 180° with stronger π-donor ligands. Presumably, the filled π-symmetry orbitals on X interact with the empty p-orbital on M, which makes this interaction energetically favorable. The results from ^1^H NMR and NIR studies show that Lewis acidity does not change greatly as the ion is varied from Al(III) to Ga(III) or when the X ligands vary. According to the measurement performed with the Gutman–Beckett method, the Lewis acidity of (I_2_P^2–^)AlX (X = Cl, H or I) and (I_2_P^2–^)GaX is lower than the Lewis acidity of other reported three- and four-coordinate Al compounds, and this presumably stems from the metalloaromatic character.

a The N_py_–M–X angle is marked as θ, and the electronic structure of the I_2_P ligand is distinct for the two geometries.

The structures and properties described in the paragraph above are consistent with metalloaromaticity. Metalloaromatic molecules are roughly defined as those with a planar structure, near-equal bond lengths for equivalent bond types, a broad and low-energy LMCT band, and negative values for the nucleus-independent chemical shift (NICS) for all nuclei in the molecule.^43^ The data we collected for four coordinate compounds in Chart 1 are consistent with a metalloaromatic structure. Theoretical studies of I_2_P and Al or Ga have reported negative NICS values, which supports the assignment of metalloaromaticity.^44^ Dianionic metalloles of Al(III) and Ga(III), as well as the tropylium cation analogues (aluminepin and gallepin), are other groups of metalloaromatic compounds in which empty atomic Al and Ga p-orbitals participate in π-bonding with planar organic ligands.^45^ SP complexes comprising a d-block metal and I_2_P ligand exhibit antiferromagnetic coupling between ligand radicals and an Fe center rather than delocalization.^46^ A comparison of group 14 and group 4 cyclopropene analogues showed that group 4 complexes are closer to classical organometallic π complexes while group 14 complexes are aromatic.^47^ These examples highlight the unique aromatic electronic properties afforded when the metal center has valence p-orbitals as in main-group coordination complexes, in contrast to the bonding and electronic structures found in d-block coordination compounds.

#### 1.3.1 Exchange Coupling and Ligand Mixed Valency

**Chart 2:**
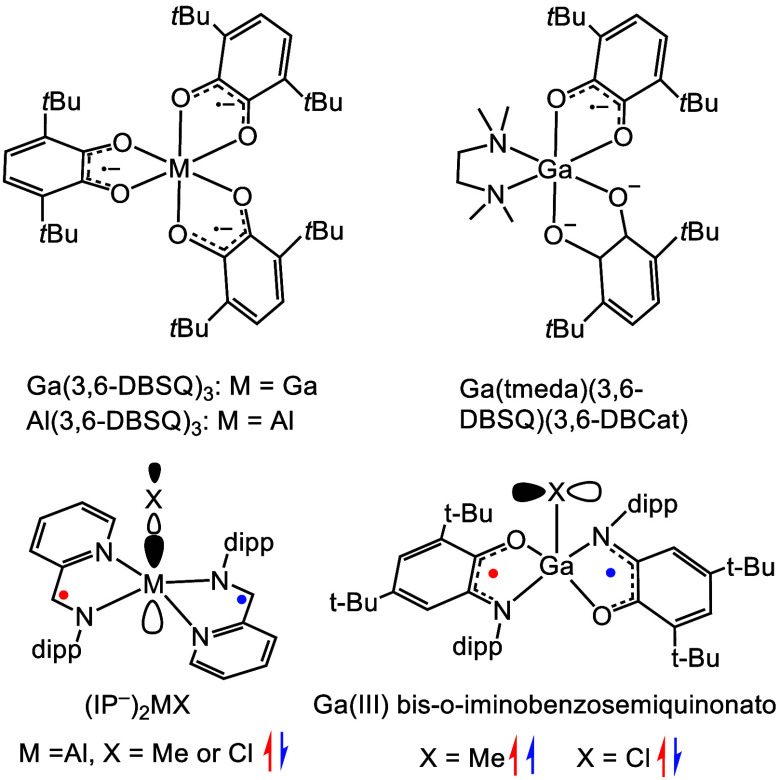
(top) Early Examples of Reduced Ligand Catecholate Complexesa and (bottom) Line Drawings of (IP^–^)_2_MX^50^ and (SQ^–^)_2_GaX^52^ with Possible Orbital Interactions Illustrated

**Chart 3:**
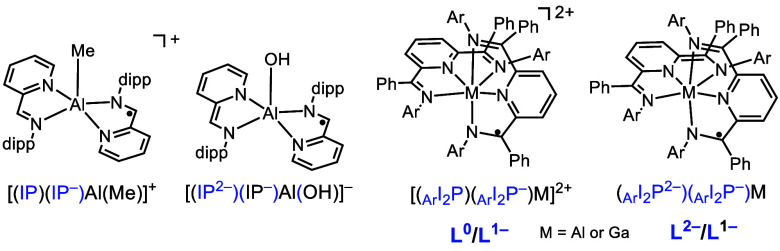
Mixed-Valent Compounds of IP (left) and I_2_P (right) Ligands

When two ligands coordinate to a single group 13 ion, unpaired electrons on the ligands can interact with each other. These interactions occur via exchange coupling in the case of two unpaired electrons or by varying degrees of delocalization for a single unpaired electron. The earliest investigation of these possibilities was reported in the work of Pierpont and co-workers with tri- and dicatecholate complexes of Al(III) and Ga(III) (Chart 2).^48^ For IP^–^ and I_2_P^–^ ligands paired with group 13 centers, magnetic exchange coupling is observed between the ligand-based unpaired electrons. For (IP^–^)_2_MX (M = Al or Ga), the exchange coupling is antiferromagnetic, and the proposed pathway for exchange is through the M–X σ* orbital. The strength of the exchange coupling in these species ranged from *J* = −230 to −370 cm^–1^ (Chart 3).^37,49−51^ Magnetic exchange coupling has also been observed between two radicals on [(_o-PhOMe_I_2_P^–^)_2_M]^+^ complexes (M = Al, Ga). Variable-temperature EPR spectroscopy revealed a net ferromagnetic interaction between the ligand radicals with *J* coupling values of 56 and 35.0 cm^–1^ for [(I_2_P^–^)_2_Al]^+^ and [(I_2_P^–^)_2_Ga]^+^, respectively. Published *J* values for *o*-iminobenzoquinone biradical complexes with group 13 metals range from −68.6 to −128 cm^–1^ for Ga complexes and from −122 to −179 cm^–1^ for Al.^52^ Coupling of octahedral tridentate diaryl amine Ga complexes display a coupling of ∼200 cm^–1^.^53^

a 3,6-DBSQ and tmeda are the chelating 3,6-di-*tert*-butylsemiquinone and tetramethylethylenediamine ligands.^48^

When two ligands bound to a metal have different charge states, there is the possibility of mixed-valent character and electron delocalization. Both [(IP)(IP^–^)Al(Me)][BAr_4_] and [(IP^2–^)(IP^–^)Al(OH)][Na(THF)(DME)]^38,49^ have localized Class I electronic structure, and that assignment was made based on the absence of an intervalence charge transfer (IVCT) band in the NIR region. Complexes (_Ar_I_2_P^2–^)(_Ar_I_2_P^–^)M are Class III delocalized when M = Al or Ga, and delocalization is lowered to Class II for M = In (Chart 3).^1,34^ We proposed that the near-octahedral coordination geometry of these I_2_P Al and Ga complexes provides an orbital pathway through metal-based p-orbitals to support electron delocalization. Delocalization effects are diminished with the larger In ion, which permits non-octahedral geometry about the central ion. In addition, delocalization is most efficient when the overall molecular charge is lowest and the fewest electrons populate the π* ligand framework. For Al(III), an analogous series of compounds was prepared with electron-withdrawing and -donating ligands, _PhF_5__I_2_P and _*p*-PhNMe_2__I_2_P, respectively, where delocalization was reduced and unchanged, respectively. Access to 5 charge states separated by one electron in octahedral I_2_P Al complexes also lends to their use as flow battery analytes which can store many electrons to achieve high charge density.^54^

## 2 Reaction Chemistry and Applications of NIL (Group 13) Complexes

This section explores the reaction chemistry and electrochemical energy storage methods that are enabled by our access to the many charge states in NIL (group 13) complexes. For ease of reference, this section has been divided into three parts: section 2.1 discusses ligand-based ET for reaction chemistry and energy storage; section 2.2 features ligand-based PT that enables catalysis via metal–ligand cooperative bond activation; and section 2.3 presents ligand-based PT coupled with ET, and this includes hydride transfer chemistry.

### 2.1 Ligand-Based ET for Reaction Chemistry

**Scheme 6:**
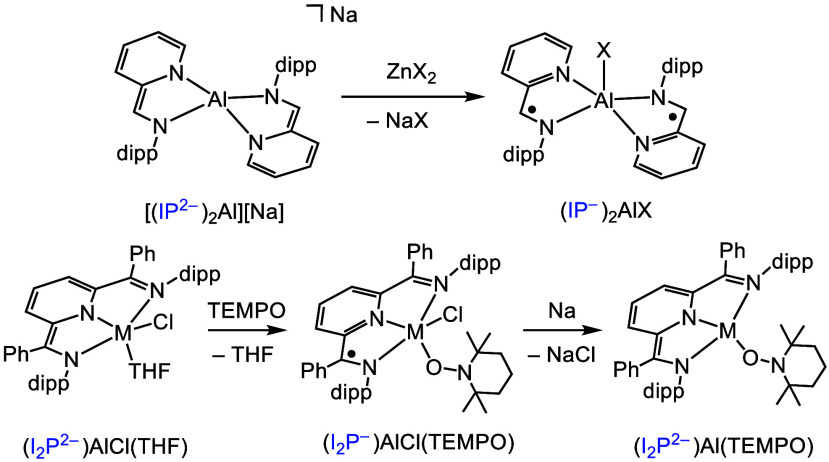
Group Transfer Chemistry of Selected IP and I_2_P Complexes X = Cl, CCPh, N_3_, SPh, and NHPh; TEMPO = (2,2,6,6-tetramethylpiperidin-1-yl)oxyl radical.

There are a number of reaction classes that traditionally proceed with the support of oxidation-state changes at a transition element center.^55,56^ One of these is group transfer chemistry, where a two-electron oxidation-state change of a metal center is concomitant with the formation of a new metal–ligand bonding interaction. This reaction type is important in biological enzymes, e.g., cytochrome P450,^57^ and in synthetic organic chemistry where epoxidation of alkenes and aziridination chemistry via N atom transfer has been widely studied. A variation of this common reaction type in NIL complexes involves the ligand supporting the ET events that enable the bond-breaking and -making events at the metal, as can be witnessed in an early report with Zr.^58,59^ The first report of this type for group 13 appeared in 2011.^50^ The oxidation of [(IP^2–^)_2_Al][Bu_4_N] by pyridine-*N*-oxide led to Al(III)–oxo intermediates, which reacted with available C–H bonds in reaction solution to give (IP^–^)_2_AlOH. In this example, ligand-centered oxidation mirrors the reactivity of first-row transition metals in the oxidation of C–H bonds; the metal–oxo species ultimately introduce C–OH functionality. In related chemistry, oxidation of [(IP^2–^)_2_Al]^−^ with ZnX_2_ salts provides reproducible 2e^–^ oxidation reactions with the Zn^0/2+^ couple, where each IP ligand is oxidized by a single electron to afford (IP^–^)_2_AlX.^50^ Along with the benefit of reproducible oxidations, the use of metal salts allows for a wide variety of X groups to be employed to form Al–X bonds. We report reactions of X = Cl, CCPh, N_3_, SPh, and NHPh (Scheme 6). Reactions of either [(IP^2–^)2Al]^−^ or [(IP^2–^)2Ga]^−^ with tetramethylthiuram disulfide and other oxidants also afford two-electron group transfer products.^51,60,61^

In I_2_P-supported Al chemistry, reaction of the (2,2,6,6-tetramethylpiperidin-1-yl)oxyl radical, TEMPO, with (I_2_P^2–^)AlCl(THF) yields (I_2_P^–^)AlCl(TEMPO). Reduction of this complex by an equivalent of Na produces (I_2_P^2–^)Al(TEMPO), providing a unique example of ligand substitution by redox cycling.^62^ Subsequent isolation of [(^H^I_2_P^3–^)Al(TEMPO)]^−^ represents a rare example of a singly ligated (I_2_P^3–^)–Al coordination complex. This was made possible by group transfer chemistry, which afforded a reductively stable Al–O interaction between the Al center and TEMPO. Ligand-based redox chemistry has also been reported with the BIAN and the ONO ligands (Scheme 3). As examples, reduction of aromatic ketones, R_2_CO, to pinacolates is reported with (BIAN^2–^)AlI(Et_2_O), where ET from BIAN^2–^ to R_2_CO produces a [(BIAN^–^)AlI]_2_[μ-O_2_C_2_R_4_] intermediate;^63^ addition of orthoquinones, quinO, to (ONO^cat^)^2–^AlCl(Et_2_O) gives the (ONO^sq^)^2–^Al(py)(quinO), in which the ONO ligand is deprotonated and oxidized by one electron.^36^

### 2.2 Ligand-Based PT: Catalysis via Metal–Ligand Cooperative Bond Activation

**Scheme 7:**
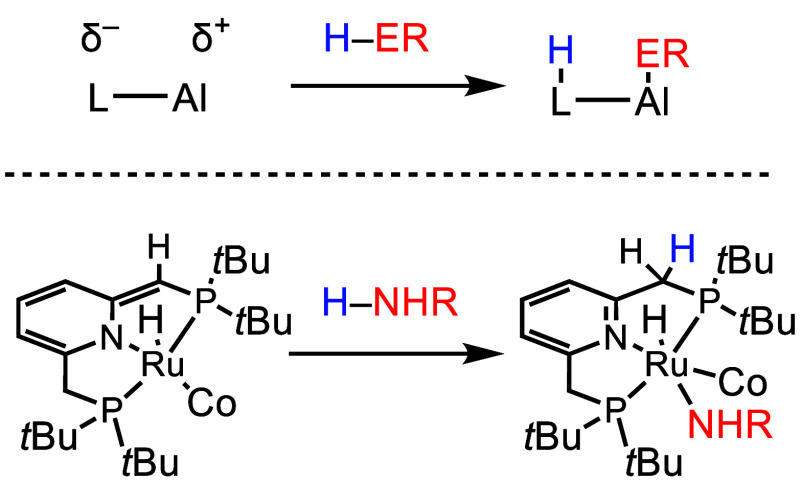
(top) General MLCBA Activation of RE–H Bond by Noninnocent Al Complexes and (bottom) MLCBA Using De-/Re-aromatization Reactivity Demonstrated by Milstein L is the multidentate ligand, R is an alkyl group, and E is typically a N or O atom whose bond E–H will be cleaved.

Having discussed ligand-based ET chemistry, we now discuss PT chemistry that is enabled by the Lewis-basic sites available in reduced NIL complexes of Al(III). Ligand O and N atoms can function as PT sites, enabling metal–ligand cooperative bond activation (MLCBA). In this reaction type, Al(III) behaves as an electrophile, and the Lewis-basic reduced ligand site is a nucleophile resulting in heterolytic bond cleavage of the substrate (Scheme 7). Metalloaromaticity serves to reduce the Lewis acidity of the formal Al(III) ion in complexes such as (I_2_P^2–^)AlH, (I_2_P^2–^)AlCl, and (I_2_P^2–^)AlI, and this feature of the electronic structure is important in promoting MLCBA instead of substrate coordination to the Al(III) ion. Polar bond activation is favorable only in some instances, such as in the work of Milstein and co-workers using Ru and Fe complexes (Scheme 7).^64^ In those examples, the MLCBA reaction results in de-/rearomatization of the catalyst-supporting ligand. Interestingly, in all of the examples of MLCBA described below with Al(III), the polar bond activation step also induces a de-/rearomatization of the catalyst-supporting ligand. Others have also recently shown that metalloaromatic group 13 compounds can exhibit MLCBA type reactivity which varies based on the nature of the substrate and the metallocycle.^65,66^

**Scheme 8:**
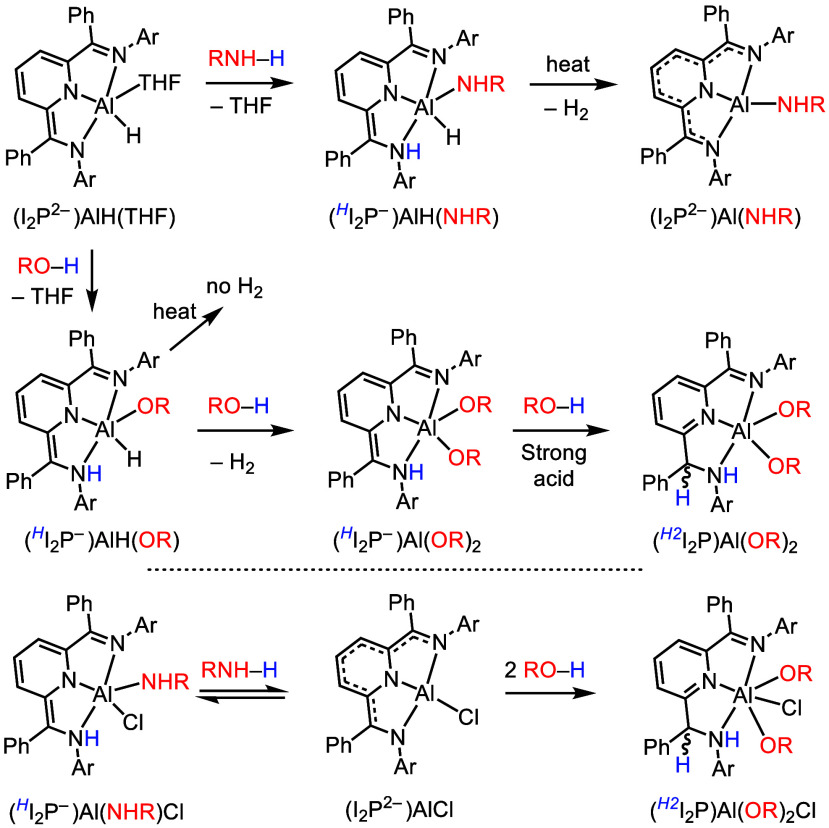
Reactivity Summary of (I_2_P^2–^)AlH(THF) and (I_2_P^2–^)AlCl with Alcohols or Amines H or H_2_ is added to the label; this denotes the ligand is once or twice protonated, respectively.

Using I_2_P^2–^ as the supporting ligand for Al(III), MLCBA toward weakly acidic RNH_2_ or ROH substrates results in O–H or N–H bond activation, respectively.^41,67−69^ As a specific example, consider the reaction of an amine or alcohol with (I_2_P^2–^)AlH(THF) (Scheme 8). The reaction of (HI_2_P^2–^)AlH(THF) with RNH_2_ or ROH affords (HI_2_P^–^)AlH(NHR) or (HI_2_P^–^)AlH(OR), respectively. Protonation of the amido group of the reduced I_2_P^2–^ ligand has occurred and is shown by an italicized superscript on the ligand label (Scheme 8).^67^ In the case where the substrate is weakly acidic such as when using RNH_2_ to produce (HI_2_P^–^)AlH(NHR), a follow-up reaction produces H_2_ and (I_2_P^2–^)Al(NHR) when mild heat is applied. The products obtained from more acidic substrates such as alcohols or formic acid, (HI_2_P^–^)AlH(OR), do not lose H_2_ even at elevated temperatures. However, (HI_2_P^–^)AlH(OR) does react with further equivalents of acid to give products of the form (HI_2_P^–^)Al(OR)_2_ with the release of H_2_. With sufficiently low p*K*_a_, a further reaction produces (H_*2*_I_2_P)Al(OR)_2_, such as when formic acid is employed (Scheme 8, second line).^41,68^ The stoichiometric MLCBA reactions described above do in some cases initiate catalysis, for example, in the dehydrogenative coupling of amines when benzylamine is the substrate^41^ and in the dehydrogenation of HCOOH.^70^

Reactions of (I_2_P^2–^)AlCl follow a different pathway after the initial MLCBA step (Scheme 8, bottom). Following MLCBA, there is no option for the release of H_2_ because the Al center does not have a hydride ligand. Reactions of (I_2_P^2–^)AlCl with alcohols including *i*PrOH or Ph_2_CH(OH) afford (^*H_2_*^I_2_P)Al(OR)_2_Cl; whereas the reaction of (I_2_P^2–^)AlCl with an amine such as aniline produces an equilibrium mixture of the substrates and (^*H*^I_2_P^–^)Al(NHR)Cl. A further difference is that (I_2_P^2–^)AlCl reacts more readily with alcohols whereas (I_2_P^2–^)AlH reacts more readily with amines. A recent report described (I_2_P^2–^)AlCl(THF) as a precatalyst for the Meerwein–Ponndorf–Verley oxidation of isopropanol to acetone.^3,71^

**Scheme 9:**
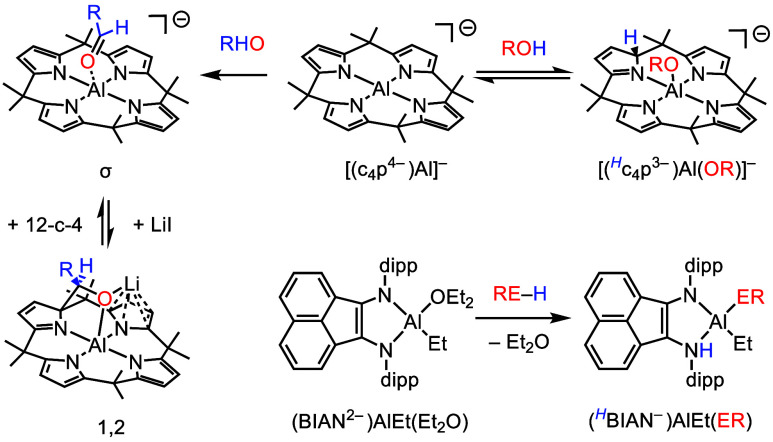
MLCBA Reactivity of [(c_4_p^4–^)Al]^−^ and (BIAN^2–^)AlEt(Et_2_O) c_4_p^4–^ = calix[4]pyrrolato. 12-c-4 = 12-crown-4. Below the c_4_p^4–^ complexes on the left, the σ and 1,2 labels denote the binding mode of the aldehyde/ketone, which is dependent on the presence of lithium cation.

Group 13 complexes with other anionic ligands have also been reported to support PT reactions. Fedushkin and co-workers have reported several MLCBA examples with (BIAN)–Al and (BIAN)–Ga complexes (BIAN = example shown in Scheme 3) for the activation of RE–H^72^ or C–C π bonds.^73^ An illustrative example of this MLCBA reactivity is the reaction of (BIAN^2–^)AlEt(Et_2_O) with amines and alcohols, RE–H (Scheme 9, bottom). This MLCBA reactivity was instrumental in the ring-opening polymerization (ROP) catalysis performed by dimeric (BIAN)Al–Al(BIAN).^74^ In another example, calix[4]pyrrolato Al complexes, [(c_4_p^4–^)Al]^−^, undergo protonation at a carbon atom of the pyrazole upon activation of the O–H bond in alcohols, ROH, where R = EtOH, *i*PrOH, *t*BuOH, 4-methylbenzyl alcohol, 4-nitrobenzyl alcohol, bis(4-methoxyphenyl)methanol 4-bromophenol, and benzoic acid (Scheme 9, top right).^75^ This selectivity, C versus N protonation, is attributed to the strained cyclic geometry of the ligand. N protonation of [(c_4_p^4–^)Al]^−^ would result in an energetically unfavorable distortion from planarity. Greb and co-workers have also shown that [(c_4_p^4–^)Al]^−^ can bind reversibly with aldehydes and ketones in a bidentate fashion to form C–C and Al–O bonds (Scheme 9, top left).^76^ Switching between the σ and 1,2-adducts was controlled by the presence or absence of a Li^+^ ion (Scheme 9, left). [(c_4_p^4–^)Al]^−^ was found to be an active catalyst for the hydroboration of 4-nitrobenzaldehyde, 4-dimethylamino benzaldehyde, and acetone with pinacolborane. Slower reaction rates were obtained for aldehydes with electron-withdrawing substituents, presumably resulting from the preference for activated aldehydes to adopt catalytically deactivated 1,2-binding. This observed rate is opposite the general trend that activated aldehydes react faster with hydrides, and it provides an exciting prospect for how substrate binding and MLCBA can influence selectivity in future reactions involving noninnocent Al complexes.

### 2.3 Ligand Proton-Coupled Electron Transfer (PCET): Hydride Transfer (HT) Chemistry

When a hydride ion is transferred to a C atom of pyridine, a dihydropyridinate (DHP^–^) is formed. The resulting DHP^–^ features a tetrahedral C with two H atoms on the C of the former pyridine ring. DHP^–^ chemistry has a long history that is primarily derived from efforts to model the organic cofactor nicotinamide adenine diphosphate (NAD^+^) which is known to be an efficient HT agent.^77^ Those model systems suffer almost universally from large overpotentials needed for the reduction of the NAD^+^ model analog to generate the NADH model equivalent via a transfer of two electrons and one proton.^78,79^ Work on pyridyl (group 13) compounds has previously taught us that the p-valence orbitals of Al and Ga have a good energy match and alignment with N-donor reduced ligands. Interactions of these p orbitals with the ligand orbitals lower the potential needed to inject multiple electrons into the I_2_P ligands featured in much of this Account. This section describes our ongoing efforts to employ this strategy of using group 13 ions with organic ligands to lower barriers to pyridine reduction and the energy for cycling through DHP^–^ intermediates for hydride transfer (HT) to substrates. Our goals in this work are 2-fold: to model the redox chemistry of NAD^+^ and to develop functional HT catalysts for applications in organic synthesis.

**Scheme 10:**
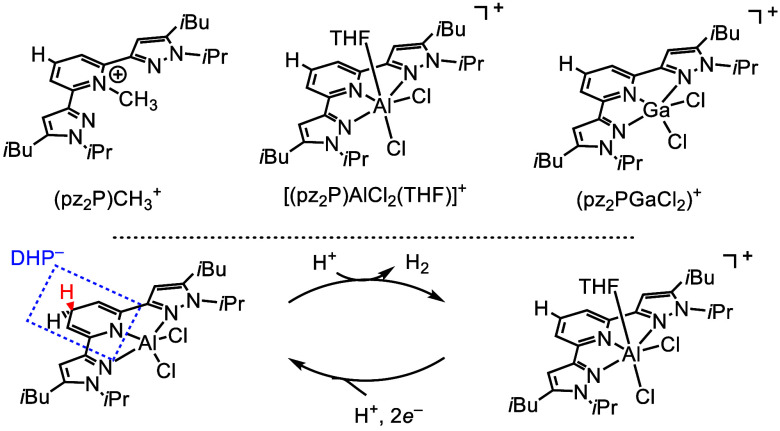
(top) Comparison of Selected pz_2_P Salts and (bottom) General Scheme for How Al Complexes Participate in HER and as HT Catalysts Dashed blue box highlights dihydropyridinate (DHP^–^).

To facilitate DHP^–^ formation, we turned to the dipyrazoylylpyridine ligand platform (pz_2_P as defined in Scheme 3 and related ligands). Weakly basic pyrazole groups on pz_2_P allow ET and PT chemistry to be localized at the pyridyl ring in group 13 complexes of pz_2_P-type ligands (Scheme 10).^80^ This is in contrast to the I_2_P chemistry discussed above where the imino donors are reduced before the pyridyl donor of the pincer ligand. In one report a structural analog to NAD^+^, [(pz_2_P)CH_3_]OTf, was synthesized by alkylating pz_2_P at the central pyridyl N. Cyclic Voltammetry (CV) of [(pz_2_P)CH_3_)]OTf displayed irreversible reduction events that have a potential *E*_p_ that is ∼900 mV more anodic relative to ligand-based reduction events of pz_2_P-type complexes of divalent metals.^81^ Experiments probing the origin of the irreversibility of the reduction event suggest the formation of DHP^–^ species: (pz_2_^H^P^–^)CH_3_, (pz_2_^H^P^–^)AlCl_2_, and (pz_2_^H^P^–^)GaCl_2_. The origin of the 900 mV anodic shift relative to the divalent metal complexes of pz_2_P was also discussed, and several contributing factors are possible. These include two main ideas: (1) (pz_2_^H^P^–^)CH_3_, (pz_2_^H^P^–^)AlCl_2_, and (pz_2_^H^P^–^)GaCl_2_ are all cations, whereas the divalent metal complexes are uncharged; and (2) AlCl_2_^+^, GaCl_2_^+^, and CH_3_^+^ interact with pz_2_P via valence p-orbitals, whereas valence d-orbitals are involved for the Zn^II^ and Fe^II^ complexes. Greater delocalization through the p-orbital manifold is likely based on our prior observations with metalloaromatic Al and Ga I_2_P complexes.

Electrocatalytic activity of [(pz_2_P)AlCl_2_(THF)]AlCl_4_ for the H_2_ evolution reaction (HER) offers insight into how complexes may function as HT catalysts (Scheme 10 bottom). Other redox-inactive metal complexes of NILs have also since been reported to exhibit HER and carbon dioxide reduction reaction (CO_2_RR). CO_2_RR behavior has been reported in work by Brudvig,^82^ Grapperhaus,^83^ Kumar,^84^ and their co-workers. Reports of catalytic HT from (DHP^–^)–Al complexes are also known, but those may proceed via Al–hydride or Al–(DHP^–^) intermediates.^85^

## 3 Summary and Outlook

This Account outlines the chemistry of Al(III) when combined with NILs. NIL–Al(III) complexes have been isolated in 6 charge states, and these have led to insights into various electronic properties of the compounds. As an example, the importance of the empty Al(III) valence p-orbitals in facilitating electronic coupling and delocalization between Al and ligand and between two ligands that are connected by a single Al center has been demonstrated. Metalloaromaticity has been used as a framework to rationalize some of the structures and reactivity properties, and probes of metalloaromatic (I_2_P^2–^)MX complexes demonstrate that more electron density in the M p-orbital leads to decreased Lewis acidity which can be used to tune future metalloaromatic complexes to control reaction pathways and substrate scope. Another material property that can be influenced by I_2_P is stability toward coordinating or protic solvents. Octahedral NIL–Al I_2_P complexes are stable in MeCN or in the presence of trace water despite highly reduced oxidation states.^54^

The unusual electronic properties of the new molecular Al complexes described above have given rise to new reaction chemistry. NIL–Al(III) complexes support ET reactions, including those where both 1 and 2 electron oxidation and reduction reactions occur. Ligand-based PT chemistry supports mechanisms following MLCBA pathways as an entry point to many reaction types.

For example, formic acid and benzyl amine dehydrogenation and transfer hydrogenation have all been demonstrated. Along with ET and PT reactivity, control of ligand p*K*_a_ values can influence the PCET vs PT reaction chemistry, ligand-based PCET chemistry offers an attractive method to lower the potentials needed to drive the formation of ligand-based C–H hydrides, and future work will expand HT reactivity of C–H hydride species. Through ligand design and correct reaction conditions, the chemistry of molecular Al continues to expand through the aid of noninnocent ligand complexes. We foresee NIL continuing to expand the chemistry of Al by providing new material properties, substrate scope, and catalytic reaction pathways.

## References

[ref1] Arnold A.; Sherbow T. J.; Sayler R. I.; Britt R. D.; Thompson E. J.; Munoz T. M.; Fettinger J. C.; Berben L. A. Organic electron delocalization modulated by ligand charge states in [L_2_M]^*n*−^ complexes of Group 13 ions. J. Am. Chem. Soc. 2019, 141, 15792–15803. 10.1021/jacs.9b05602. 31510741

[ref2] Bass T. M.; Carr C. R.; Sherbow T. J.; Fettinger J. C.; Berben L. A. Synthesis of Square Planar Gallium Complexes and a Proton NMR Correlation Probing Metalloaromaticity. Inorg. Chem. 2020, 59, 13517–13523. 10.1021/acs.inorgchem.0c01908. 32883068

[ref3] Carr C. R.; Vesto J. I.; Xing X.; Fettinger J. C.; Berben L. A. Aluminum–ligand cooperative O–H Bond Activation Initiates Catalytic Transfer Hydrogenation. ChemCatChem. 2022, 14, e202101869 10.1002/cctc.202101869.

[ref4] Power P. Main-Group Elements as Transition Metals. Nature 2010, 463, 171–177. 10.1038/nature08634. 20075912

[ref5] Weetman C.; Inoue S. The Road Travelled: After Main-Group Elements as Transition Metals. ChemCatChem. 2018, 10, 4213–4228. 10.1002/cctc.201800963.

[ref6] Yaroshevsky A. A. Abundances of Chemical Elements in the Earth’s Crust. Geochem. Int. 2006, 44, 48–55. 10.1134/S001670290601006X.

[ref7] Primary Commodity Prices, Actual Market Prices for Non-Fuel and Fuel Commodities, 2018–2023. International Monetary Fund, 2023, https://www.imf.org/en/Research/commodity-prices (accessed 2023-07-03).

[ref8] Liu Y.; Li J.; Ma X.; Yang Z.; Roesky H. W. The Chemistry of Aluminum(I) With β-Diketiminate Ligands and Pentamethylcyclopentadienyl-Substituents: Synthesis, Reactivity and Applications. Coord. Chem. Rev. 2018, 374, 387–415. 10.1016/j.ccr.2018.07.004.

[ref9] Ochiai T.; Franz D.; Inoue S. Applications of N-heterocyclic Imines in Main Group Chemistry. Chem. Soc. Rev. 2016, 45, 6327–6344. 10.1039/C6CS00163G. 27499203

[ref10] Li B.; Yang Y.; Zhu H.; Roesky H. W. (β-Diketiminato)Aluminum Hydroxides and the Chalcogenide Derivatives: Precursors for Homo- And Heterometallic Complexes With Al-E-M (E = Chalcogen, M = Metal) Frameworks. Coord. Chem. Rev. 2021, 429, 213625 10.1016/j.ccr.2020.213625.

[ref11] Franz D.; Inoue S. Cationic Complexes of Boron and Aluminum: An Early 21st Century Viewpoint. Chem.—Eur. J. 2019, 25, 2898–2926. 10.1002/chem.201803370. 30113744

[ref12] Li W.; Ma X.; Walawalkar M. G.; Yang Z.; Roesky H. W. Soluble Aluminum Hydrides Function as Catalysts in Deprotonation, Insertion, and Activation Reactions. Coord. Chem. Rev. 2017, 350, 14–29. 10.1016/j.ccr.2017.03.017.

[ref13] Ni C.; Ma X.; Yang Z.; Roesky H. W. Recent Advances in Aluminum Compounds for Catalysis. Eur. J. Inorg. Chem. 2022, 2022, e202100929 10.1002/ejic.202100929.

[ref14] Jung H.-J.; Cho Y.; Kim D.; Mehrkhodavandi P. Cationic Aluminum, Gallium, and Indium Complexes in Catalysis. Catal. Sci. Technol. 2021, 11, 62–91. 10.1039/D0CY01741H.

[ref15] Dagorne S.; Wehmschulte R. Recent Developments on the Use of Group 13 Metal Complexes in Catalysis. ChemCatChem. 2018, 10, 2509–2520. 10.1002/cctc.201800045.

[ref16] Gualandi A.; Calogero F.; Potenti S.; Cozzi P. G. Al(Salen) Metal Complexes in Stereoselective Catalysis. Molecules 2019, 24, 1716 10.3390/molecules24091716. 31052604 PMC6540592

[ref17] Goldsmith C. R. Aluminum and Gallium Complexes as Homogeneous Catalysts for Reduction/Oxidation Reactions. Coord. Chem. Rev. 2018, 377, 209–224. 10.1016/j.ccr.2018.08.025.

[ref18] Greb L.; Ebner F.; Ginzburg Y.; Sigmund L. M. Element–Ligand Cooperativity with p-Block Elements. Eur. J. Inorg. Chem. 2020, 2020, 3030–3047. 10.1002/ejic.202000449.

[ref19] Berben L. A. Catalysis by aluminum(III) complexes of non-innocent ligands. Chem.—Eur. J. 2015, 21, 2734–2742. 10.1002/chem.201405400. 25429760

[ref20] Zhong M.; Sinhababu S.; Roesky H. W. The Unique β-diketiminate Ligand in Aluminum(I) and Gallium(I) Chemistry. Dalton Trans. 2020, 49, 1351–1346. 10.1039/C9DT04763H. 31942579

[ref21] Zhu H.; Chai J.; Jancik V.; Roesky H. W.; Merrill W. A.; Power P. P. The Selective Preparation of an Aluminum Oxide and Its Isomeric C–H-Activated Hydroxide. J. Am. Chem. Soc. 2005, 127, 10170–10171. 10.1021/ja052400y. 16028919

[ref22] Lyaskovskyy V.; de Bruin B. Redox Non-Innocent Ligands: Versatile New Tools to Control Catalytic Reactions. ACS Catal. 2012, 2, 270–279. 10.1021/cs200660v.

[ref23] Luca R. O.; Crabtree R. H. Redox-Active Ligands in Catalysis. Chem. Soc. Rev. 2013, 42, 1440–1459. 10.1039/C2CS35228A. 22975722

[ref24] Cole B. E.; Wolbach J. P.; Dougherty W. G. Jr.; Piro N. A.; Kassel W. S.; Graves C. R. Synthesis and Characterization of Aluminum-α-diimine Complexes over Multiple Redox States. Inorg. Chem. 2014, 53, 3899–3906. 10.1021/ic5003989. 24660986

[ref25] Scott J.; Gambarotta S.; Korobkov I.; Knijnenburg Q.; de Bruin B.; Budzelaar P. H. M. Formation of a Paramagnetic Al Complex and Extrusion of Fe during the Reaction of (Diiminepyridine)Fe with AlR3 (R = Me, Et). J. Am. Chem. Soc. 2005, 127, 17204–17206. 10.1021/ja056135s. 16332066

[ref26] Geoffrey F.; Cloke N.; Dalby C. I.; Henderson M. J.; Hitchcock P. B.; Kennard C. H. L.; Lamb R. N.; Raston C. L. Paramagnetic Aluminium-l,4-Di-t-butyl-l,4-diazabutadiene (dbdab) Complexes Derived from Metal Vapours and/or Metal Hydrides: Crystal Structures of [Al(dbdab)2] and [Al(dbdab){N(But)CH2}2]. J. Chem. Soc., Chem. Commun. 1990, 1394 10.1039/C39900001394.

[ref27] Romelt C.; Weyhermuller T.; Wieghardt K. Structural Characteristics of Redox-Active Pyridine-1,6-Diimine Complexes: Electronic Structures and Ligand Oxidation Levels. Coord. Chem. Rev. 2019, 380, 287–317. 10.1016/j.ccr.2018.09.018.

[ref28] Jurca T.; Dawson K.; Mallov I.; Burchell T.; Yap G. P. A.; Richeson D. S. Disproportionation and Radical Formation in The Coordination of “GaI” With Bis(Imino)Pyridines. Dalton Trans. 2010, 39, 1266–1272. 10.1039/B920047A. 20104353

[ref29] Chirik P. J.; Wieghardt K. Radical Ligands Confer Nobility on Base-Metal Catalysts. Science 2010, 327, 794–795. 10.1126/science.1183281. 20150476

[ref30] Chirik P. J. Iron- and Cobalt-Catalyzed Alkene Hydrogenation: Catalysis with Both Redox-Active and Strong Field Ligands. Acc. Chem. Res. 2015, 48, 1687–1695. 10.1021/acs.accounts.5b00134. 26042837

[ref31] Clyburne J. A. C.; Culp R. D.; Kamepalli S.; Cowley A. H.; Decken A. Ring Systems Containing Anionic and Cationic Gallium Centers: Structural and Bonding Considerations. Inorg. Chem. 1996, 35, 6651–6655. 10.1021/ic960647d. 11666825

[ref32] Lukoyanov A. N.; Fedushkin I. L.; Hummert M.; Schumann H. Aluminum Complexes with Mono- and Dianionic Diimine Ligands. Russ. Chem. Bull. 2006, 55, 422–428. 10.1007/s11172-006-0273-4.

[ref33] Thompson E. T.; Myers T. W.; Berben L. A. Synthesis of square-planar Al(III) complexes. Angew. Chem., Int. Ed. 2014, 53, 14132–14134. 10.1002/anie.201407098. 25318847

[ref34] Arnold A.; Sherbow T. J.; Bohanon A. M.; Sayler R. I.; Britt D. R.; Smith A. J.; Fettinger J. C.; Berben L. A. Delocalization tunable by ligand substitution in [L2Al]n- complexes highlights a mechanism for strong electronic coupling. Chem. Sci. 2021, 12, 675–682. 10.1039/D0SC02812F. PMC8179017 34163799

[ref35] Phan N. A.; Fettinger J. C.; Berben L. A. A Ligand Protonation Series in Aluminum(III) Complexes of Tridentate Bis(enol)amine Ligand. Inorg. Chem. 2018, 37, 4527–4533. 10.1021/acs.organomet.8b00628.

[ref36] Szigethy G.; Heyduk A. F. Aluminum Complexers of the Redox-Active [ONO] Pincer Ligand. Dalton Trans. 2012, 41, 8144–8152. 10.1039/c2dt30295k. 22669327

[ref37] Myers T. W.; Kazem N.; Stoll S.; Britt D. R.; Shanmugam M.; Berben L. A. A Redox Series of Complexes: Characterization of Four Oxidation States Including a Ligand Biradical State Stabilized via Exchange Coupling. J. Am. Chem. Soc. 2011, 133, 8662–8672. 10.1021/ja2015718. 21568319

[ref38] Myers T. W.; Yee G. M.; Berben L. A. Redox-induced carbon–carbon bond formation by using noninnocent ligands. Eur. J. Inorg. Chem. 2013, 2013, 3831–3835. 10.1002/ejic.201300192.

[ref39] Myers T. W.; Berben L. A. A Sterically Demanding Iminopyridine Ligand Affords Redox-Active Complexes of Aluminum(III) and Gallium(III). Inorg. Chem. 2012, 51, 1480–1488. 10.1021/ic201729b. 22220939

[ref40] Fedushkin I. L.; Moskalev M. V.; Lukoyanov A. N.; Tishkina A. N.; Baranov E. V.; Abakumov G. A. Dialane with a Redox-Active Bis-amido Ligand: Unique Reactivity towards Alkynes. Chem.—Eur. J. 2012, 18, 11264–11276. 10.1002/chem.201201364. 22847958

[ref41] Myers T. W.; Berben L. A. Aluminum–Ligand Cooperative N–H Bond Activation and an Example of Dehydrogenative Coupling. J. Am. Chem. Soc. 2013, 135, 9988–9990. 10.1021/ja4032874. 23799284

[ref42] Phan N. A.; Sherbow T. J.; Fettinger J. C.; Berben L. A. Synthesis of Unsupported Primary Phosphido Complexes of Aluminum(III*)*. Z. Anorg. Allg. Chem. 2021, 647, 1824–1829. 10.1002/zaac.202100199.

[ref43] Kysliak O.; Schreiner S. H. F.; Grabicki N.; Liebing P.; Weigend F.; Dumele O.; Kretschmer R. A Planar Five-Membered Aromatic Ring Stabilized by Only Two π-Electrons. Angew. Chem., Int. Ed. 2022, 61, e202206963 10.1002/anie.202206963. PMC9401857 35593009

[ref44] Li W.; Lyu Y.; Zhang H.; Zhu M.; Tang H. A theoretical study on the unusual square-planar structure of bis(imino)pyridine-ligated Group 13 complexes. Dalton Trans. 2017, 46, 106–115. 10.1039/C6DT03775E. 27900379

[ref45] Ota K.; Kinjo R. Heavier Element-Containing Aromatics of [4n+2]-Electron Systems. Chem. Soc. Rev. 2021, 50, 10594–10673. 10.1039/D0CS01354D. 34369490

[ref46] Bart S. C.; Chlopek K.; Bill E.; Bouwkamp M. W.; Lobkovsky E.; Neese F.; Wieghardt K.; Chirik P. J. Electronic Structure of Bis(imino)pyridine Iron Dichloride, Monochloride, and Neutral Ligand Complexes: A Combined Structural, Spectroscopic, and Computational Study. J. Am. Chem. Soc. 2006, 128, 13901–13912. 10.1021/ja064557b. 17044718

[ref47] Aysin R. R.; Leites L. A.; Bukalov S. S. Aromaticity of 1-Heterocyclopropenes Containing an Atom of Group 14 or 4. Organometallics 2020, 39, 2749–2762. 10.1021/acs.organomet.0c00351.

[ref48] Lange C. W.; Conklin B. J.; Pierpont C. G. Radical Superexchange in Semiquinone Complexes Containing Diamagnetic Metal Ions. 3,6-Di-tert-butyl-l,2-Semiquinonate Complexes of Zinc(II), Cobalt(III), Gallium(III), and Aluminum (III). Inorg. Chem. 1994, 33, 1276–1283. 10.1021/ic00085a012.

[ref49] Myers T. W.; Berben L. A. Countercations Direct One- or Two-Electron Oxidation of an Al(III) Complex and Al(III)Oxo Intermediates Activate CH Bonds. J. Am. Chem. Soc. 2011, 133, 11865–11867. 10.1021/ja203842s. 21774452

[ref50] Myers T. W.; Holmes A. L.; Berben L. A. Redox routes to substitution of aluminum(III): synthesis and characterization of (IP^–^)_2_AlX (IP = α-iminopyridine, X = Cl, Me, SMe, S_2_CNMe_2_, C≡CPh, N_3_, SPh, NHPh). Inorg. Chem. 2012, 51, 8997–9004. 10.1021/ic301128m. 22839746

[ref51] Kowolik K.; Shanmugam M.; Myers T. W.; Cates C. D.; Berben L. A. A redox series of gallium(III) complexes: ligand-based two-electron oxidation affords a gallium–thiolate complex. Dalton Trans. 2012, 41, 7969–7976. 10.1039/c2dt30112a. 22426475

[ref52] Ershova I. V.; Bogomyakov A. S.; Fukin G. K.; Piskunov A. V. Features of Magnetic Behavior in the Row of Pentacoordinated Bis-o-Iminobenzosemiquinonato Metal (Al, Ga, In) Complexes. Eur. J. Inorg. Chem. 2019, 2019, 938–948. 10.1002/ejic.201801348.

[ref53] Liddle B. J.; Wanniarachchi S.; Hewage J. S.; Lindeman S. V.; Bennett B.; Gardinier J. R. Electronic Communication Across Diamagnetic Metal Bridges: A Homoleptic Gallium(III) Complex of a Redox-Active Diarylamido Based Ligand and Its Oxidized Derivatives. Inorg. Chem. 2012, 51, 12720–12728. 10.1021/ic301437f. 23163736 PMC3601749

[ref54] Arnold A.; Dougherty R. J.; Carr C. R.; Reynolds L. C.; Fettinger J. C.; Augustin A.; Berben L. A. A Stable Organo-Aluminum Analyte Enables Multielectron Storage for a Nonaqueous Redox Flow Battery. J. Phys. Chem. Lett. 2020, 11, 8202–8207. 10.1021/acs.jpclett.0c01761. 32897076

[ref55] Baek Y.; Betley T. A. Catalytic C–H Amination Mediated by Dipyrrin Cobalt Imidos. J. Am. Chem. Soc. 2019, 141, 7797–7806. 10.1021/jacs.9b01262. 31016975 PMC7256963

[ref56] Groves J. T.; Quinn R. Aerobic Epoxidation of Olefins with Ruthenium Porphyrin Catalysts. J. Am. Chem. Soc. 1985, 107, 5790–5792. 10.1021/ja00306a029.

[ref57] Davydov R.; Kofman V.; Fujii H.; Yoshida T.; Ikeda-Saito M.; Hoffman B. M. Catalytic Mechanism of Heme Oxygenase through EPR and ENDOR of Cryoreduced Oxy-Heme Oxygenase and Its Asp 140 Mutants. J. Am. Chem. Soc. 2002, 124, 1798–1808. 10.1021/ja0122391. 11853459

[ref58] Haneline M. R.; Heyduk A. F. C-C Bond-Forming Reductive Elimination from a Zirconium(IV) Redox-Active Ligand Complex. J. Am. Chem. Soc. 2006, 128, 8410–8411. 10.1021/ja061107a. 16802801

[ref59] Stanciu C.; Jones M. E.; Fanwick P. E.; Abu-Omar M. M. Multi-electron Activation of Dioxygen on Zirconium(IV) to Give an Unprecedented Bisperoxo Complex. J. Am. Chem. Soc. 2007, 129, 12400–12401. 10.1021/ja075396u. 17887680

[ref60] Cates C. D.; Myers T. W.; Berben L. A. (IP)_2_Ga^III^ Complexes Facilitate Net Two-Electron Redox Transformations (IP = α-Iminopyridine). Organometallics 2012, 51, 11891–11897. 10.1021/ic301792w. 23092354

[ref61] Myers T. W.; Berben L. A. Redox active aluminium(III) complexes convert CO_2_ into MgCO_3_ or CaCO_3_ in a synthetic cycle using Mg or Ca metal. Chem. Commun. 2013, 49, 4175–4177. 10.1039/C2CC37208H. 23175190

[ref62] Sherbow T. J.; Parsons L. W. T.; Phan N. A.; Fettinger J. C.; Berben L. A. Ligand Conjugation Directs the Formation of a 1,3- Dihydropyridinate Regioisomer. Inorg. Chem. 2020, 59, 17614–17619. 10.1021/acs.inorgchem.0c02847. 33215919

[ref63] Fedushkin I. L.; Lukoyanov A. N.; Fukin G. K.; Hummert M.; Schumann H. Reduction of Aromatic Ketones with the (dpp-BIAN)AlI(Et2O) Complex. Russ. Chem. Bull. 2006, 55, 1177–1183. 10.1007/s11172-006-0396-7.

[ref64] Gunanathan C.; Milstein D. Metal-Ligand Cooperation by Aromatization-Dearomatization: A New Paradigm in Bond Activation and “Green” Catalysis. Acc. Chem. Res. 2011, 44, 588–602. 10.1021/ar2000265. 21739968

[ref65] Liu L. L.; Zhou J.; Cao L. L.; Stephan D. W. Phosphaaluminirenes: Synthons for Main Group Heterocycles. J. Am. Chem. Soc. 2019, 141, 16971–16982. 10.1021/jacs.9b09330. 31557435

[ref66] Drescher R.; Lin S.; Hofmann A.; Lenczyk C.; Kachel S.; Krummenacher I.; Lin Z.; Braunschweig H. Ring expansion of alumoles with organic azides: selective formation of six-membered aluminum– nitrogen heterocycles. Chem. Sci. 2020, 11, 5559–5564. 10.1039/D0SC02032J. 32874499 PMC7444475

[ref67] Sherbow T. J.; Carr C. R.; Saisu T.; Fettinger J. C.; Berben L. A. Insight into Varied Reaction Pathways for O–H and N–H Bond Activation by Bis(imino)pyridine Complexes of Al(III). Organometallics 2016, 35, 9–14. 10.1021/acs.organomet.5b00743.

[ref68] Thompson E. J.; Berben L. A. Electrocatalytic Hydrogen Production by an Aluminum(III) Complex: Ligand-Based Proton and Electron Transfer. Angew. Chem., Int. Ed. 2015, 54, 11642–11646. 10.1002/anie.201503935. 26249108

[ref69] Myers T. W.; Berben L. A. Aluminum–Amido-Mediated Heterolytic Addition of Water Affords an Alumoxane. Organometallics 2013, 32, 6647–6649. 10.1021/om400556s.

[ref70] Myers T. W.; Berben L. A. Aluminium–Ligand Cooperation Promotes Selective Dehydrogenation of Formic Acid to H_2_ and CO_2_. Chem. Sci. 2014, 5, 2771–2777. 10.1039/C4SC01035C.

[ref71] Cohen R.; Graves C. R.; Nguyen S. T.; Martin J. M. L.; Ratner M. A. The Mechanism of Aluminum-Catalyzed Meerwein-Schmidt-Ponndorf-Verley Reduction of Carbonyls to Alcohols. J. Am. Chem. Soc. 2004, 126, 14796–14803. 10.1021/ja047613m. 15535705

[ref72] Moskalev M. V.; Lukoyanov A. N.; Baranov E. V.; Fedushkin I. L. Unexpected Reactivity of an Alkylaluminum Complex of a Non-Innocent 1,2-Bis[(2,6 Diisopropylphenyl)Imino] Acenaphthene Ligand (Dpp-Bian). Dalton Trans. 2016, 45, 15872–15878. 10.1039/C6DT01750A. 27301590

[ref73] Fedushkin I. L.; Moskalev M. V.; Baranov E. V.; Abakumov G. A. Addition of Diphenylacetylene and Methylvinylketone to Aluminum Complex of Redox-Active Diimine Ligand. J. Organomet. Chem. 2013, 747, 235–240. 10.1016/j.jorganchem.2013.06.032.

[ref74] Kazarina O. V.; Gourlaouen C.; Karmazin L.; Morozov A. G.; Fedushkin I. L.; Dagorne S. Low Valent Al(II)–Al(II) Catalysts as Highly Active Ε-Caprolactone Polymerization Catalysts: Indication of Metal Cooperativity Through DFT Studies. Dalton Trans. 2018, 47, 13800–13808. 10.1039/C8DT02614A. 30106082

[ref75] Sigmund L. M.; Greb L. Reversible OH-Bond Activation And Amphoterism by Metal–Ligand Cooperativity of Calix[4]Pyrrolato Aluminate. Chem. Sci. 2020, 11, 9611–9616. 10.1039/D0SC03602A. 34094227 PMC8161688

[ref76] Ebner F.; Sigmund L. M.; Greb L. Metal–Ligand Cooperativity of the Calix[4]pyrrolato Aluminate: Triggerable C–C Bond Formation and Rate Control in Catalysis. Angew. Chem., Int. Ed. 2020, 59, 17118–17124. 10.1002/anie.202007717. PMC7540271 32573936

[ref77] Fukuzumi S.; Lee Y.-M.; Nam W. Catalytic recycling of NAD(P)H. J. Inorg. Biochem. 2019, 199, 110777 10.1016/j.jinorgbio.2019.110777. 31376683

[ref78] Ilic S.; Alherz A.; Musgrave C. B.; Glusac K. D. Importance of Proton-Coupled Electron Transfer in Cathodic Regeneration of Organic Hydrides. Chem. Commun. 2019, 55, 5583–5586. 10.1039/C9CC00928K. 31020295

[ref79] Ilic S.; Alherz A.; Musgrave C. B.; Glusac K. D. Thermodynamic and Kinetic Hydricities of Metal-Free Hydrides. Chem. Soc. Rev. 2018, 47, 2809–2836. 10.1039/C7CS00171A. 29543931

[ref80] Sherbow T. J.; Fettinger J. C.; Berben L. A. Control of Ligand p*K*a Values Tunes the Electrocatalytic Dihydrogen Evolution Mechanism in a Redox-Active Aluminum(III) Complex. Inorg. Chem. 2017, 56, 8651–8660. 10.1021/acs.inorgchem.7b00230. 28402654

[ref81] Parsons L. W. T.; Fettinger J. C.; Berben L. A. Group 13 Ion Coordination to Pyridyl Models NAD+ Reduction Potential. Chem. Commun. 2023, 59, 9110–9113. 10.1039/D3CC02562D. 37403698

[ref82] Wu Y.; Jiang J.; Weng Z.; Wang M.; Broere D. L. J.; Zhong Y.; Brudvig G. W.; Feng Z.; Wang H. Electroreduction of CO_2_ Catalyzed by a Heterogenized Zn–Porphyrin Complex with a Redox-Innocent Metal Center. ACS Cent. Sci. 2017, 3, 847–852. 10.1021/acscentsci.7b00160. 28852698 PMC5571454

[ref83] Cronin S. P.; Mamun A. A.; Toda M. J.; Mashuta M. S.; Losovyj Y.; Kozlowski P. M.; Buchanan R. M.; Grapperhaus C. A. Utilizing Charge Effects and Minimizing Intramolecular Proton Rearrangement to Improve the Overpotential of a Thiosemicarbazonato Zinc HER Catalyst. Inorg. Chem. 2019, 58, 12986–12997. 10.1021/acs.inorgchem.9b01912. 31503487

[ref84] Upadhyay A.; Saurav K. V.; Varghese E. L.; Hodage A. S.; Paul A.; Awasthi M. K.; Singh S. K.; Kumar S. Proton Reduction by a Bimetallic Zinc Selenolate Electrocatalyst. RSC Adv. 2022, 12, 3801–3808. 10.1039/D1RA08614F. 35425408 PMC8981091

[ref85] Zhang G.; Wu J.; Zeng H.; Neary M. C.; Devany M.; Zheng S.; Dub P. A. Dearomatization and Functionalization of Terpyridine Ligands Leading to Unprecedented Zwitterionic Meisenheimer Aluminum Complexes and Their Use in Catalytic Hydroboration. ACS Catal. 2019, 9, 874–884. 10.1021/acscatal.8b04096.

